# Next‐Generation Probiotics: Emerging Mechanisms, Therapeutic Innovations, and Future Perspectives

**DOI:** 10.1155/bmri/8353908

**Published:** 2026-11-14

**Authors:** Shivam Agarwal, Yenamandra S. Prabhakar, Praveen Katiyar, Baijnath Das, Souvik Sur, Himanshu Kumar, Bina Pani Gupta

**Affiliations:** ^1^ Teerthanker Mahaveer University Moradabad Uttar Pradesh India tmu.ac.in; ^2^ CSIR-Central Drug Research Institute Lucknow Uttar Pradesh India cdri.res.in; ^3^ School of Health Sciences Chhatrapati Shahu Ji Maharaj University Kanpur Uttar Pradesh India; ^4^ Department of Medical Laboratory Science IIMT College of Medical Sciences (Allied Health Sciences) IIMT University Meerut Uttar Pradesh India; ^5^ Department of Allied Health Sciences Manipal Tata Medical College Manipal Academy of Higher Education Manipal India manipal.edu

**Keywords:** engineered probiotics, microbial therapeutics, next-generation probiotics, precision probiotics, probiotic efficacy gap

## Abstract

Probiotic research is shifting from the empirical use of broadly beneficial microorganisms toward functionally defined microbial therapeutics. Advances in microbiome science, multiomics, formulation technologies, and synthetic biology have expanded the field from conventional strains to next‐generation organisms, engineered microbes, and defined microbial effectors. This review critically examines this transition by linking mechanisms of probiotic action with the barriers that determine clinical translation. Although probiotics can influence ecological, metabolic, epithelial, immune, and host‐signaling pathways, biological activity alone does not ensure therapeutic benefit. We define this disconnect as the probiotic efficacy gap, arising when strain or function selection, delivery, functional engagement, host responsiveness, or clinical outcomes fail to align. Emerging strategies—including protective formulations, mucosa‐targeted delivery, programmable microbial platforms, nonviable microbial products, and microbiome‐guided patient selection—address distinct points of failure along this translational pathway. We further examine the manufacturing, potency, safety, biocontainment, and regulatory challenges associated with increasingly complex microbial products. Future progress will require a function‐first framework that integrates mechanism‐linked potency measures, prospective responder identification, targeted delivery, and clinically meaningful evidence of target engagement. Rather than seeking a universally effective probiotic, the field must determine which microbial function is required, how and where it should be delivered, and in which host it is most likely to produce benefit.

## 1. Introduction

Probiotics are defined as “live microorganisms that, when administered in adequate amounts, confer a health benefit on the host” [1]. This definition requires both microbial viability and evidence of a health benefit and therefore distinguishes probiotics from nonviable microbial preparations and isolated microbial products. Over the past decade, probiotic research has increasingly moved beyond empirical administration toward strain‐ and function‐specific evaluation, supported by advances in microbiome profiling, microbial genomics, metabolomics, and other multiomics approaches. Importantly, probiotic efficacy cannot be assumed from taxonomic identity alone; clinical effects may differ according to the strain, indication, dose, formulation, and study population [2]. The therapeutic landscape has also expanded beyond conventional probiotic strains. Recent studies and reviews have examined next‐generation probiotics (NGPs), including candidate commensal organisms with potentially disease‐relevant functions, as well as genetically engineered microorganisms, defined microbial consortia, and precision microbiome interventions [3–5]. Postbiotics have emerged as a separate class and are defined by the International Scientific Association for Probiotics and Prebiotics as preparations of inanimate microorganisms and/or their components that confer a health benefit on the host [6]. These categories are related through their use of microbial biology but are not scientifically or regulatorily interchangeable. In particular, nonviable preparations and purified microbial effectors should not be classified as probiotics, whereas engineered live microorganisms developed as therapeutic products may require development and regulatory pathways that differ from those applied to conventional probiotic foods or supplements [1, 6]. Despite these advances, translation of microbial activity into reproducible clinical benefit remains difficult. Experimental evidence may demonstrate antimicrobial activity, epithelial‐barrier modulation, metabolite production, immune signaling, or other biological functions without establishing that the same mechanism produces a clinically meaningful effect in humans. Moreover, administration of the same probiotic preparation does not necessarily produce uniform gastrointestinal persistence or biological responses across individuals. Human studies have demonstrated person‐ and strain‐specific patterns of mucosal colonization associated with baseline host and microbiome characteristics, supporting the importance of interindividual variation in determining probiotic responses [7]. Thus, microbial functional capacity alone is insufficient to predict therapeutic efficacy; the baseline microbial ecosystem, host biological context, delivery characteristics, ecological competition, and the ability of an intervention to achieve sustained or transient target engagement may all influence whether an individual behaves as a responder or nonresponder.

For the purposes of this review, we define the “probiotic efficacy gap” as the disconnect between evidence that a microbial intervention possesses a potentially beneficial biological function and evidence that administration produces a reproducible, clinically meaningful benefit in the intended human population. We operationalize this gap across five interconnected domains: (i) strain or product identity and functional competence; (ii) delivery, viability, and exposure at the intended site of action; (iii) compatibility with the recipient′s baseline microbiome and host biological context; (iv) demonstrable functional or target engagement in vivo; and (v) improvement in an appropriately defined clinical endpoint. Failure or substantial variability at any stage can prevent mechanistic activity from translating into clinical efficacy. Within this framework, responder–nonresponder heterogeneity is therefore considered part of the translational pathway rather than simply unexplained variation in clinical outcomes. At the mechanistic level, probiotics can interact with the host and resident microbiota through several pathways, including modulation of microbial community interactions, competitive effects against pathogens, effects on epithelial‐barrier function, production or modification of bioactive metabolites, and interactions with mucosal immune pathways [8]. However, the extent to which individual mechanisms have been demonstrated in humans varies substantially by strain and indication. Mechanistic plausibility, evidence of target engagement, and evidence of patient benefit should therefore be evaluated as distinct levels of evidence rather than treated as equivalent indicators of therapeutic efficacy (Figure 1).

**Figure 1:**
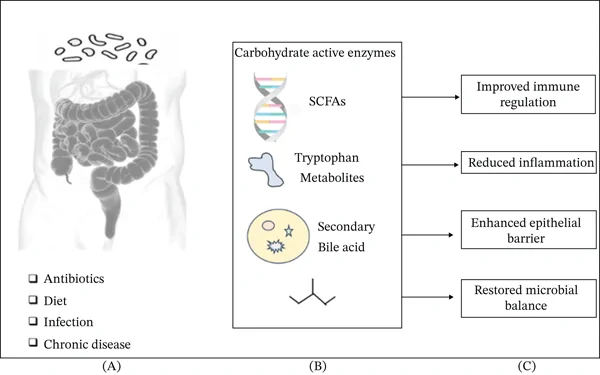
Mechanistic pathways through which probiotics mediate health effects. (A) Gut–microbiota ecosystem. (B) Functional mechanism of probiotic. (C) Host level outcomes.

Recent reviews have already described individual aspects of NGPs, engineered probiotics, synthetic‐biology approaches, postbiotics, and personalized microbiome interventions. The present review therefore extends beyond a descriptive overview of emerging probiotic technologies. It integrates mechanistic evidence, therapeutic innovation, host–microbiome variability, delivery strategies, clinical translation, safety, manufacturing, and regulatory considerations within the probiotic efficacy‐gap framework. Particular emphasis is placed on distinguishing established human evidence from emerging or preclinical findings and on evaluating how next‐generation microbial therapeutics may progress from biological plausibility toward more reproducible and precision‐oriented clinical applications. This approach provides the basis for examining the emerging mechanisms, therapeutic innovations, translational challenges, and future perspectives of NGPs.

## 2. Methodology

A structured literature search was conducted using PubMed/MEDLINE, Scopus, and Web of Science Core Collection to identify relevant peer‐reviewed literature on conventional probiotics, NGPs, engineered microbial therapeutics, postbiotics, microbial‐delivery approaches, and precision microbiome interventions. ScienceDirect and Google Scholar were used for supplementary searching, and reference lists of relevant studies and reviews were screened to identify additional publications. The literature considered in this review spans publications from 2006 to 2026. Search terms covered microbial therapeutic platforms and their mechanistic, clinical, and translational aspects, including “probiotic,” “next‐generation probiotic,” “live biotherapeutic product,” “engineered probiotic,” “postbiotic,” “gut microbiota,” “intestinal barrier,” “immune modulation,” “engraftment,” “clinical efficacy,” “responder,” “microbial delivery,” “synthetic biology,” “multiomics,” “safety,” and “regulation.” Boolean operators AND and OR were used, with database‐specific syntax. The detailed search strategies are provided in Table S1. Peer‐reviewed clinical, observational, review, translational, animal, and in vitro studies relevant to the scope of the review were considered. Publications unrelated to microbial therapeutics, duplicate reports, conference abstracts, editorials, opinion articles, and unsupported commercial sources were excluded. The study‐selection process involved identification of relevant publications through database and supplementary searches, removal of duplicate records, screening of titles and abstracts, and assessment of potentially eligible full‐text articles. Publications that met the predefined eligibility criteria and were relevant to the review objectives were retained for narrative synthesis. A total of 99 references were included in the final bibliography, covering original research, clinical studies, systematic reviews, and relevant regulatory and methodological literature. The selected evidence was synthesized narratively, with emphasis on mechanistic findings, therapeutic innovations, clinical applications, safety considerations, and translational challenges. The selected literature was synthesized narratively, with clinical evidence distinguished from mechanistic and preclinical findings where appropriate. Particular attention was given to microbial strain or product identity, host and microbiome variability, delivery, biological engagement, and clinical translation within the proposed probiotic efficacy‐gap framework. No formal risk‐of‐bias or evidence‐quality assessment was performed. Consequently, this review represents a narrative synthesis rather than a systematic evaluation of the certainty of evidence, and its conclusions should be interpreted in light of potential study‐selection bias and differences in methodological quality among the included publications.

### 2.1. From Conventional Probiotics to Next‐Generation Microbial Therapeutics

Probiotic research has gradually moved beyond the traditional use of microorganisms selected mainly because of historical use and general health benefits. Greater attention is now being given to strains with clearly defined biological functions and clinically relevant properties. Conventional probiotics commonly include members of the Lactobacillaceae family and the genus *Bifidobacterium*, along with selected *Bacillus* species and *Saccharomyces* strains [9]. Their appropriate use depends on strain‐level identification, demonstrated safety in the intended population, maintenance of viability at an effective dose, and evidence of a health benefit [10]. Clinical effects, however, cannot be generalized across species or genera, as efficacy may differ considerably between strains and clinical indications [2]. Differences in dose, formulation, host characteristics, baseline microbiome composition, and disease status may further influence the observed response. Advances in metagenomics, culturomics, metabolomics, and improved microbial cultivation methods have expanded the range of microorganisms that can be investigated for therapeutic use [11, 12]. This has led to growing interest in NGPs derived from the human microbiome. Organisms such as *Akkermansia muciniphila* and *Faecalibacterium prausnitzii* are being studied because of their specific biological functions and associations with host physiology rather than a long history of use as conventional probiotics [13]. The evidence for individual NGP candidates, however, remains uneven. Findings from experimental or preclinical studies should therefore be distinguished from evidence demonstrating consistent clinical benefit in humans. At the same time, precision probiotic strategies are being developed to account for differences between individuals in baseline microbiome composition, host phenotype, microbial interactions, and treatment response [14]. Multiomics and computational approaches may help identify host and microbial features associated with differential probiotic responsiveness and, in turn, support more individualized intervention strategies [5]. This is particularly relevant to the probiotic efficacy gap, since an intervention that produces a measurable effect in one individual or population may not generate the same level of biological engagement or clinical benefit in another.

Synthetic biology has further broadened the scope of microbial therapeutics by allowing microorganisms to be engineered for defined functions. These functions may include sensing disease‐associated signals or producing therapeutic metabolites, enzymes, and other bioactive molecules within the host [15]. Such engineered living therapeutics should be regarded as a distinct therapeutic platform rather than simply as an advanced form of conventional probiotics. Their development also raises additional considerations related to genetic stability, biocontainment, manufacturing consistency, safety, environmental release, and regulatory oversight [16].

The current microbiome‐therapeutics landscape therefore includes related but scientifically distinct platforms and approaches, including conventional probiotics, microbiome‐derived NGPs, precision probiotic strategies, and engineered living therapeutics. Their main characteristics, developmental basis, current level of evidence, and key translational considerations are summarized in Table 1.

**Table 1:** Comparison of conventional probiotics and emerging microbial therapeutic platforms.

Therapeutic paradigm	Basis of development	Representative examples	Defining therapeutic strategy	Principal translational challenge
Conventional probiotics	Historical use, established safety, strain‐level characterization, and technological suitability	*Lacticaseibacillus rhamnosus* GG; *Bifidobacterium* spp.; *Saccharomyces boulardii*	Modulation of microbial ecology, epithelial barrier function, and host–microbe interactions	Variable and strain‐ and indication‐specific clinical efficacy. [2, 10]
Next‐generation probiotics	Microbiome‐based identification of commensal microorganisms with defined biological functions	*Akkermansia muciniphila*; *Faecalibacterium prausnitzii*	Restoration or augmentation of specific microbial, metabolic, or host‐associated functions	Cultivation and formulation challenges, oxygen sensitivity of some candidate strains, and limited clinical validation. [13]
Precision probiotic strategies	Selection or tailoring of microbial interventions according to microbiome, host, metabolic, and clinical characteristics	Individually selected strains or defined microbial combinations	Matching microbial functions with host‐specific biological characteristics and predicted treatment response	Limited availability of validated responder biomarkers and standardized patient‐selection frameworks. [5]
Engineered living therapeutics	Synthetic biology and rational genetic engineering of microorganisms	Engineered *Escherichia coli* Nissle 1917; modified *Lactococcus lactis*	Detection of disease‐associated signals and controlled in situ production of therapeutic molecules	Genetic stability, biocontainment, manufacturing consistency, safety, environmental release, and regulatory complexity [15, 16].

### 2.2. Mechanisms of Action: From Microbial Activity to Host Response

Probiotic activity arises from interactions among the administered strain, resident microbiota, intestinal environment, and host rather than from a single biological pathway [17]. At the intestinal level, probiotics may compete with other microorganisms for nutrients and adhesion sites, produce antimicrobial compounds, alter the local metabolic environment, and interact with epithelial and immune cells [8, 18]. These effects can influence microbial ecology, epithelial‐barrier function, and mucosal immune responses. However, the contribution of individual mechanisms varies among strains and biological settings, and demonstration of a mechanistic effect does not, by itself, establish clinical efficacy. Within the microbial ecosystem, competitive exclusion can occur through competition for nutrients or attachment sites and through the production of organic acids, bacteriocins, and other inhibitory compounds. These activities may reduce pathogen adhesion, growth, or colonization under appropriate experimental conditions [18]. Importantly, sustained intestinal colonization is not necessarily required for every probiotic effect; functional activity during gastrointestinal transit may also contribute to host–microbe interactions (Figure 2). The biological relevance of such effects nevertheless depends on the strain, exposure, microbial environment, and host context [19–21]. At the mucosal interface, probiotic cells, surface‐associated structures, and microbial metabolites can interact with epithelial and immune pathways [22]. Probiotic‐derived or microbiota‐modified compounds, including short‐chain fatty acids, indole‐related metabolites, organic acids, exopolysaccharides, and other microbial products, have been associated with changes in mucus production, epithelial junctional regulation, immune signaling, and intestinal homeostasis [22, 23]. Some of these metabolites are produced directly by administered microorganisms, whereas others arise through metabolic interactions with resident members of the microbiota. This distinction is important because the metabolic effect observed after probiotic administration may reflect a broader microbial network rather than the activity of the administered strain alone. Microbial products and cell‐associated signals may also influence intracellular pathways involved in inflammation and epithelial responses [24]. Experimental studies have described modulation of signaling pathways including NF‐*κ*B, MAPK, and PI3K/Akt in response to selected probiotic strains or their products [25]. Such of this evidence is mechanistic and derived from cell‐based or animal models, and the extent to which pathway modulation occurs at comparable levels in humans remains strain‐ and indication‐dependent. Similarly, microbial metabolites may influence processes beyond the intestine through immune, metabolic, neural, or endocrine signaling [26], but evidence of systemic biological activity should be distinguished from demonstrated improvement in patient‐level clinical outcomes. These mechanisms can therefore be viewed as three interacting axes: microbial ecosystem modulation, metabolite‐mediated barrier.

**Figure 2:**
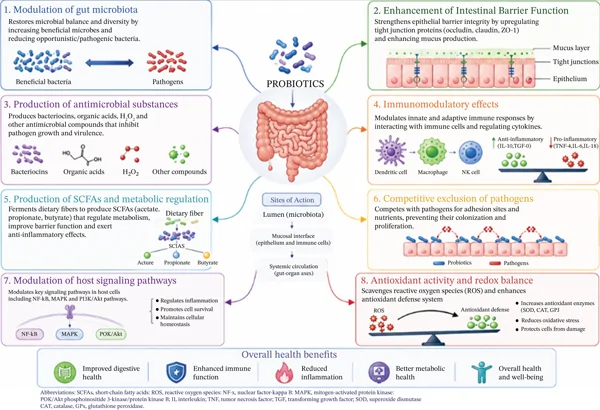
Integrated mechanisms of probiotic action at the microbiota–intestinal barrier–host interface.

Their clinical relevance is shaped by strain identity, formulation and exposure, baseline microbiome composition, host phenotype, and disease context. This mechanistic heterogeneity forms an important component of the probiotic efficacy gap, as biological activity observed at one level does not necessarily progress to measurable target engagement or reproducible clinical benefit. The major mechanistic axes and their principal translational uncertainties are summarized in Table 2.

**Table 2:** Major mechanistic axes of probiotic action and their translational evidence.

Mechanistic axis	Principal processes and mediators	Main biological target	Potential functional effect	Evidence context	Major translational question
Microbial ecosystem modulation and pathogen exclusion	Competition for nutrients and adhesion sites; organic acids; bacteriocins and other inhibitory compounds	Resident microbiota and enteric pathogens	Reduced pathogen adhesion, growth, colonization, or fitness	Strong mechanistic and experimental evidence; clinical relevance remains strain‐ and indication‐specific	Whether transient ecological changes are sufficient to produce reproducible clinical benefit
Metabolite‐mediated barrier and immune regulation	SCFAs, indole‐related metabolites, organic acids, exopolysaccharides, and metabolites generated through microbial cross‐feeding	Intestinal epithelium and mucosal immune system	Modulation of mucus production, epithelial‐junction function, immune signaling, and local homeostasis	Supported predominantly by mechanistic and translational studies, with human evidence varying by metabolite and intervention	Which strain–metabolite–host relationships are reproducible and clinically relevant in humans
Host cellular signaling and systemic communication	Microbial metabolites and cell‐associated signals influencing NF‐*κ*B, MAPK, PI3K/Akt, and related pathways	Epithelial and immune‐cell signaling networks	Potential modulation of inflammatory, epithelial‐survival, and redox‐related responses	Much of the pathway‐specific evidence remains experimental; direct clinical attribution is limited	Whether pathway modulation occurs at biologically meaningful exposure levels and contributes to patient‐level outcomes

### 2.3. From Mechanistic Promise to Therapeutic Innovation

The clinical evidence for probiotics is not uniform across therapeutic indications. Among gastrointestinal applications, the most consistent human evidence has been reported for the prevention of antibiotic‐associated diarrhea and for probiotics used alongside standard *Helicobacter pylori* eradication therapy, although the magnitude of benefit remains dependent on the microbial preparation, dose, background treatment, and study population [27, 28]. Irritable bowel syndrome represents a more heterogeneous setting: Pooled randomized evidence indicates improvement in selected symptoms, but considerable variation in strains, formulations, treatment duration, and outcome assessment makes it difficult to attribute these effects to a single probiotic intervention [29]. Evidence from metabolic and neuropsychiatric applications illustrates a different stage of translation. In type 2 diabetes, probiotic supplementation has been associated with modest changes in glycemic markers, but available trials do not establish whether these short‐term metabolic effects translate into reductions in long‐term clinical complications [30]. Similarly, studies evaluating probiotics for depression and anxiety have produced inconsistent findings across symptom scales and formulations, suggesting that host characteristics, intervention composition, and outcome selection may influence treatment response [31]. These observations support a more cautious interpretation of mechanistic or biomarker changes when patient‐level benefits have not been consistently demonstrated. Site‐directed microbial interventions provide useful examples of how therapeutic effects may become more clearly linked to a defined microorganism and anatomical target. Vaginal administration of *Lactobacillus crispatus* CTV‐05 has shown strain‐specific efficacy in reducing recurrence of bacterial vaginosis following standard antibiotic treatment [32]. In periodontal disease, probiotic supplementation has also been investigated as an adjunct to conventional nonsurgical therapy, with several studies reporting improvements in selected periodontal measures; however, differences in strain selection, dosing schedules, and study design continue to limit identification of an optimal regimen [33, 34]. Taken together, these findings indicate that probiotic translation should be evaluated at the level of the specific strain or formulation, clinical indication, comparator, and outcome, rather than from taxonomic identity or mechanistic plausibility alone. The strength of current human evidence, representative interventions, and major limitations across selected clinical applications are summarized in Table 3.

**Table 3:** Selected human clinical evidence for probiotic interventions across therapeutic areas.

Clinical application	Intervention(s) represented	Evidence type	Key quantitative finding	Interpretation and limitation
Antibiotic‐associated diarrhea	Multiple preparations; significant subgroup signals reported for *Saccharomyces boulardii*, *Lactobacillus acidophilus*, *L. casei*, *L. paracasei*, *L. rhamnosus*, *Bifidobacterium longum*, and selected *Bacillus* spp.	Systematic review and meta‐analysis; 42 RCTs, 11,305 adults	Overall AAD risk: RR 0.63 (95% CI 0.54–0.73). *S. boulardii*: RR 0.63 (0.46–0.86); *B. longum*: RR 0.46 (0.28–0.73)	Supports a preventive effect overall, but effects differed by microbial species, dose and baseline AAD risk; results should not be generalized to every probiotic product [27]
*Helicobacter pylori* eradication adjunct	Single‐ and multistrain preparations; no single strain‐specific pooled intervention	Umbrella review of 28 meta‐analyses based on 534 RCTs	Eradication: RR 1.10 (95% CI 1.06–1.14); total adverse effects: RR 0.54 (95% CI 0.42–0.70)	Supports adjunctive use with standard eradication therapy, not probiotic monotherapy. Most underlying meta‐analyses were rated critically low quality [28]
Irritable bowel syndrome	Multiple single‐ and multistrain preparations; *Lactobacillus*, *Bifidobacterium*, *Bacillus*, *Saccharomyces*, and other preparations represented across trials	Three‐level meta‐analysis; 72 RCTs	Global IBS symptoms: SMD −0.55 (95% CI −0.76 to −0.34); abdominal pain: SMD −0.89 (−1.29 to −0.50); QoL: SMD 0.99 (0.45–1.54)	Short‐term pooled benefit was observed, but very high heterogeneity and variation among formulations prevent attribution to a single strain [29]
Type 2 diabetes mellitus	Mainly *Lactobacillus*‐ and *Bifidobacterium*‐containing single or mixed formulations; no single strain accounts for the pooled estimate	Systematic review and meta‐analysis; 30 RCTs, 1827 participants	FBG: SMD −0.331 (95% CI −0.424 to −0.238); HbA1c: SMD −0.421 (−0.584 to −0.258); HOMA‐IR: SMD −0.224 (−0.342 to −0.105)	Supports modest improvement in glycaemic biomarkers; does not establish reduction in long‐term diabetic complications. Considerable intervention heterogeneity remains [30]
Depressive symptoms/anxiety	Mixed formulations containing species such as *Lactobacillus acidophilus*, *L. paracasei*, *L. casei*, *L. plantarum*, *L. salivarius*, *Bifidobacterium bifidum*, *B. breve*, and *B. longum*	Meta‐analysis; 12 RCTs, 707 participants	BDI: MD −2.69 (95% CI −4.22 to −1.16); HAMD: MD −1.40 (−3.29 to 0.48), not significant; DASS and MADRS also not significant	Findings differ according to symptom scale and formulation; evidence does not support a uniform psychobiotic effect [31]
Recurrent bacterial vaginosis	*Lactobacillus crispatus* CTV‐05 (Lactin‐V) following vaginal metronidazole	Phase 2b randomized, double‐blind placebo‐controlled trial; *n* = 228	Recurrence at Week 12: 30% vs. 45%; RR 0.66 (95% CI 0.44–0.87), *p* = 0.01. CTV‐05 detected in 79% at Week 12	Strong direct strain‐specific human evidence, although broader clinical generalization requires additional confirmation [32]
Periodontitis—adjunctive probiotic therapy	Predominantly *Limosilactobacillus/Lactobacillus reuteri*‐based interventions; an illustrative RCT used DSM 17938 + ATCC PTA 5289, 1 × 10^8^ CFU of each strain per lozenge	Systematic review/network meta‐analysis; supported by strain‐specific RCTs	Network meta‐analysis found significant PPD effects for nine interventions, CAL effects for 18, and BOP effects for eight. In the strain‐specific RCT, greater PPD reduction and CAL gain occurred in moderate/deep pockets (*p* < 0.05)	Possible adjunctive effect, but intervention heterogeneity and inconsistent trial‐level findings prevent identification of a universally effective regimen [33, 34]

### 2.4. The Probiotic Efficacy Gap: Why Biological Activity Does not Ensure Clinical Benefit

The heterogeneous clinical findings summarized in Table 3 illustrate a central problem in probiotic translation: Biological activity alone does not guarantee a clinically meaningful response. A microorganism may possess a relevant functional property, yet its effect can be lost or weakened during delivery [35]. Processing and storage conditions, gastric acidity, bile exposure, intestinal transit, and competition with resident microorganisms can all influence the viable and functional exposure ultimately achieved at the intended site of action [21]. Thus, successful strain selection represents only the first step in the pathway from microbial activity to clinical benefit. The next challenge is ecological compatibility within the recipient microbiome. Whether an administered microorganism persists or functions in the intestine depends on the properties of the strain, available ecological niches, the resident microbial community, and the host environment [36]. Durable engraftment, however, should not be considered an absolute requirement for probiotic activity. A microorganism may remain transient yet still exert a functional effect during its passage through the gastrointestinal tract [21]. For this reason, the relevant translational question is not simply whether a strain colonizes the host, but whether sufficient exposure results in the intended biological or functional engagement. Host‐related variability adds a further layer of complexity. In a randomized probiotic intervention among adults with metabolic syndrome, population‐wide improvement was not observed, whereas a subgroup of participants showed favorable changes in selected metabolic measures; responders and nonresponders also differed in microbiome characteristics and dietary patterns [37]. Baseline microbiome composition, together with host metabolic and immune characteristics, has therefore become an important consideration in attempts to understand and predict differential responses to microbiota‐directed interventions [38]. These observations support the inclusion of responder–nonresponder heterogeneity within the efficacy‐gap framework rather than attributing variable outcomes solely to the administered microorganism. In this review, the probiotic efficacy gap is not intended to present strain selection, delivery, microbiome compatibility, or host variability as newly recognized barriers. Instead, it organizes these established determinants into a sequential translational framework consisting of six checkpoints: (i) appropriate strain or function selection, (ii) adequate delivery and exposure, (iii) ecological compatibility or sufficient transient activity, (iv) functional target engagement, (v) host responsiveness, and (vi) achievement of a clinically meaningful outcome (Figure 3). This structure helps identify where translation may fail and what type of evidence is required at each stage. Importantly, a change in microbial abundance, metabolite concentration, inflammatory marker, or other biomarker should not automatically be interpreted as therapeutic success, because biomarker modification does not necessarily predict improvement in how a patient feels, functions, or experiences disease [39]. The framework therefore separates microbial activity, target engagement, host response, and clinical benefit, providing a practical basis for evaluating why apparently biologically active interventions may succeed in some settings but fail in others.

**Figure 3:**
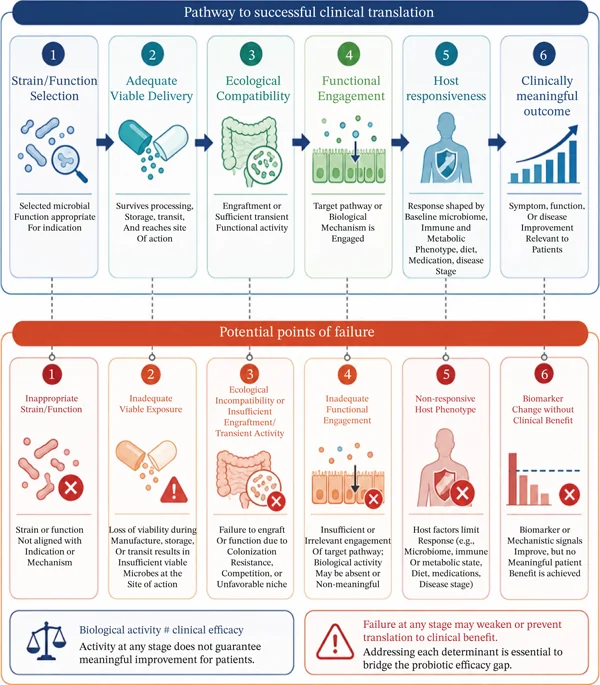
The probiotic efficacy gap: determinants of successful clinical translation.

### 2.5. Engineering the Intervention: Overcoming the Probiotic Efficacy Gap

The efficacy gap outlined in Figure 3 can partly be addressed by improving the design and delivery of the microbial intervention. A biologically appropriate strain must remain sufficiently viable during manufacturing, storage, administration, and gastrointestinal transit before it can exert its intended function at the target site. Delivery therefore represents an important component of the translational pathway, alongside ecological compatibility, functional engagement, and host responsiveness [21, 36]. Protective formulation, surface modification, rational microbial design, and engineered microbial platforms are being investigated to improve control over these different stages of translation [13, 14, 33].

### 2.6. Overcoming Viability and Delivery Failure

The biological activity of a probiotic depends not only on the functional properties of the strain but also on maintaining an adequate viable and functional exposure during processing, storage, and gastrointestinal passage [40]. Heat, oxygen, moisture, gastric acidity, bile, and other environmental stresses can reduce microbial survival before the intended site of action is reached [41]. Consequently, a strain that performs well under experimental conditions may show reduced activity in vivo if the delivered formulation does not provide sufficient protection [42].

Microencapsulation is widely investigated as a means of reducing these losses [43]. Probiotic cells can be entrapped or coated within protective matrices designed to limit exposure to adverse processing and gastrointestinal conditions and, in some formulations, to facilitate release at a defined intestinal site [41, 44]. Commonly investigated materials include alginate, chitosan, pectin, starch and other polysaccharides, protein‐based matrices such as whey or casein, and lipid‐based carriers and their performance is strongly influenced by the microbial strain, composition of the matrix, particle characteristics, processing conditions, and intended release environment [45, 46].

The method used to prepare the formulation is equally important. Spray drying is attractive because of its scalability and comparatively efficient processing, but thermal exposure can reduce cell viability [45]. Freeze drying generally avoids high‐temperature exposure but requires longer processing and may increase production costs [47]. Extrusion, emulsification, coacervation, multilayer coating, and related approaches provide additional options for balancing microbial survival, storage stability, manufacturing feasibility, and controlled release [48–50]. Importantly, improved viable counts during storage or simulated gastrointestinal testing should be regarded primarily as delivery‐performance evidence; they do not by themselves demonstrate therapeutic efficacy in patients. Synbiotic formulations provide another strategy for modifying the microbial environment. According to the ISAPP consensus, a synbiotic comprises live microorganisms together with substrate(s) selectively utilized by host microorganisms that confer a health benefit. Depending on the formulation, the substrate may complement or function synergistically with the administered microorganisms [51].

Human evidence for encapsulated formulations requires cautious interpretation. Clinical studies may demonstrate that a formulation is well tolerated or associated with improvement in selected outcomes, but the contribution of encapsulation cannot be inferred unless the study directly compares otherwise equivalent encapsulated and unencapsulated interventions. For example, a randomized trial in irritable bowel syndrome evaluated a combination containing probiotics, microencapsulated sodium butyrate, and short‐chain fructooligosaccharides; although improvement was reported in selected gastrointestinal outcomes, the design does not permit the clinical effect to be attributed specifically to microencapsulation or to the probiotic component alone [52]. Table 4 therefore distinguishes evidence related to probiotic viability, gastrointestinal delivery, and microbial recovery from evidence of patient‐level clinical efficacy.

**Table 4:** Human evidence relevant to probiotic formulation and microencapsulation.

Formulation approach	Study design and population	Intervention and comparator	Evidence assessed	Main finding	Interpretation
Encapsulated single‐strain probiotic	Whorwell et al.; multicentre, randomized, double‐blind, placebo‐controlled trial; 362 women with IBS	*Bifidobacterium longum* subsp. *infantis* 35624 at 1 × 10^6^, 1 × 10^8^, or 1 × 10^10^ CFU/day for 4 weeks versus placebo	Clinical efficacy of the final encapsulated product	The 1 × 10^8^ CFU/day dose improved abdominal pain/discomfort and several other IBS symptoms compared with placebo; the lower and higher doses did not show the same pattern of benefit	Demonstrates dose‐ and formulation‐specific clinical activity, but does not establish a benefit attributable to encapsulation because no equivalent unencapsulated formulation was evaluated [53]
Microencapsulated versus uncoated probiotic strains	Del Piano et al.; double‐blind, randomized crossover study; 44 healthy volunteers	*Lactiplantibacillus plantarum* LP01 (LMG P‐21021) + *Bifidobacterium breve* BR03 (DSM 16604); microencapsulated formulation at 1 × 10^9^ CFU/strain/day versus uncoated formulation at 5 × 10^9^ CFU/strain/day	Gastrointestinal delivery and fecal recovery	Both formulations increased fecal *Lactobacilli* and *Bifidobacteria*. The lower dose microencapsulated preparation produced recovery kinetics comparable with the fivefold higher dose uncoated preparation	Provides direct human evidence relevant to delivery efficiency and intestinal recovery, but not to therapeutic efficacy. Because administered doses differed, the study also does not provide a same‐dose comparison of encapsulation alone [54]
Multicomponent formulation containing probiotics and microencapsulated butyrate	Gąsiorowska et al.; randomized, double‐blind, placebo‐controlled trial; 120 adults with Rome IV‐defined IBS	Five probiotic strains combined with colon‐targeted microencapsulated sodium butyrate and short‐chain fructooligosaccharides for 12 weeks versus placebo	Clinical efficacy of a combined formulation	Adequate symptom relief at Week 4 was reported by 64.7% of the intervention group versus 42.0% receiving placebo (*p* = 0.023); no significant between‐group differences were observed for IBS‐SSS or IBS‐QOL	Supports clinical activity of the combined product, but the independent contribution of the probiotic strains, microencapsulated butyrate, prebiotic substrate, or encapsulation strategy cannot be determined [52]

### 2.7. Improving Spatial Retention and Functional Engagement

Successful gastrointestinal delivery does not necessarily ensure functional engagement if an administered microorganism is rapidly cleared or fails to reach the intended biological interface [55]. Recent preclinical work has therefore explored hyaluronic acid (HA)‐containing coatings and delivery systems that act directly on probiotic microorganisms rather than relying on evidence from unrelated HA applications. These studies show that HA‐containing platforms can contribute to protection during gastrointestinal transit, surface engineering, site‐directed delivery, and maintenance of microbial activity [56–58]. However, the available evidence remains preclinical, and improved viability, localization, or intestinal abundance should not be interpreted as established therapeutic efficacy in humans. The translational value of spatial‐retention technologies will ultimately depend on whether improved local exposure produces reproducible target engagement and clinically meaningful benefit (Table 5).

**Table 5:** Preclinical evidence for HA‐containing probiotic delivery and functional.

HA‐based strategy	Microbial intervention	Experimental setting	Main finding	Evidence interpretation
HA–poly(propylene sulfide) nanoparticle–probiotic platform	*Escherichia coli* Nissle 1917 (EcN)	Gastrointestinal‐stress experiments, ex vivo intestinal adhesion, and DSS‐induced colitis in mice	HA‐PPS nanoparticles were conjugated to modified EcN, whereas a poly‐norepinephrine surface layer improved oral viability and intestinal retention. The combined platform showed greater activity than unmodified EcN in experimental colitis	Direct microbial‐delivery evidence involving an HA‐containing platform. Importantly, the enhanced mucoadhesion and prolonged retention were primarily attributed to the poly‐norepinephrine coating, not to HA alone; clinical efficacy has not been established [58]
Hyaluronic‐acid‐methacrylate/DNA microcapsules	*Escherichia coli* Nissle 1917	Simulated gastrointestinal transit and experimental inflammatory bowel disease models	HA‐methacrylate‐containing microcapsules protected probiotic viability during gastrointestinal passage, migrated toward inflamed colonic regions, and enabled microenvironment‐responsive bacterial release	Provides direct evidence for HA‐containing protection and site‐directed probiotic delivery, but findings remain preclinical [57]
HA‐modified probiotic surface with prebound nutrient nanoparticles	*Lacticaseibacillus rhamnosus* GG ATCC 53103 and *Escherichia coli* Nissle 1917	In vitro microbial competition/GI‐stress experiments and murine colitis	Probiotic surfaces were modified with HA and assembled with engineered ginger‐derived nanoparticles. The system improved resistance to gastrointestinal stress, nutrient uptake, proliferation, and intestinal probiotic abundance in mice	Demonstrates direct HA‐based probiotic surface engineering and improved functional fitness; it does not establish human efficacy or prove that HA alone accounts for the observed effects [56]

### 2.8. Programming Therapeutic Function

Synthetic biology allows selected microorganisms to be equipped with defined sensing and therapeutic functions, including detection of local disease‐associated signals and controlled production or modification of biologically active molecules [59, 60]. Engineered live bacterial therapeutics have therefore been developed as platforms in which microbial fitness, circuit behavior, therapeutic output, and host exposure must be considered together rather than as independent design features [61]. Increasing genetic complexity can also create additional failure points, including metabolic burden [62], loss of circuit function, inappropriate activation, and reduced fitness under conditions that differ substantially from simplified laboratory systems.

Biosafety is particularly important for engineered microorganisms. Horizontal gene transfer, environmental dissemination, and unintended persistence may increase risk when recombinant genetic material is introduced into living therapeutic strains [63]. Proposed containment approaches include auxotrophic dependencies, conditional survival systems, and synthetic kill switches, although escape through mutation or acquisition of compensating genetic functions remains possible and therefore requires experimental assessment [64, 65]. These safeguards should be evaluated alongside microbial shedding, genetic stability, and persistence under physiologically and environmentally relevant conditions.

Clinical translation further depends on reproducible identity, potency, viability, genetic stability, and batch‐to‐batch performance. Engineered microorganisms intended to prevent or treat disease should therefore be evaluated as therapeutic biological products rather than assumed to follow the same development pathway as conventional probiotic foods or supplements [66]. Analytical characterization, manufacturing control, stability, and clinically relevant measures of potency remain central challenges in live biotherapeutic product (LBP) development [67]. The major failure modes and corresponding validation requirements are summarized in Table 6.

**Table 6:** Failure‐oriented design and validation framework for engineered living therapeutics.

Design domain	Main potential failure	Required control/design consideration	Key validation parameter	Translational interpretation
Microbial chassis	Reduced fitness, unpredictable host interaction, or inappropriate persistence	Selection of a genetically tractable organism with suitable physiological fitness and defined safety characteristics	Growth kinetics, metabolic burden, competitive fitness, host interaction, and persistence	A suitable chassis should retain the engineered function under host‐relevant conditions without unacceptable disturbance of the resident microbiota [61]
Disease sensing and therapeutic output	False activation, failure to detect the intended signal, or inadequate/excessive payload production	Sensors and effector modules matched to physiologically relevant signal concentrations and therapeutic requirements	Detection threshold, specificity, dynamic range, response time, payload concentration, and biological activity	Demonstration of sensing or payload production in vitro is insufficient unless the complete input–output response remains functional in vivo [61]
Genetic and phenotypic stability	Mutation, circuit silencing, expression drift, or fitness loss during prolonged growth	Stable circuit architecture with limited metabolic burden and appropriate genomic integration where feasible	Genetic integrity across generations, expression variability, leakiness, and maintenance of therapeutic function	Programmed activity should remain reproducible during manufacturing, storage, administration, and host exposure [61, 68]
Horizontal gene transfer and environmental dissemination	Transfer of engineered DNA to other microorganisms, shedding, or persistence outside the intended host environment	Reduction of transferable genetic elements and assessment of environmental survival and dissemination	Gene‐transfer potential, duration of shedding, survival outside the host, and recovery of engineered genetic material	HGT and environmental release require direct biosafety evaluation rather than being treated only as theoretical risks [65]
Biocontainment	Escape of viable engineered organisms or failure of the containment mechanism	Auxotrophy, synthetic nutrient dependence, kill switches, conditional survival systems, or redundant containment strategies	Escape frequency, kill‐switch failure, mutation rate, containment stability, and survival under stress	Biocontainment can reduce risk but does not eliminate it; evolutionary escape and circuit failure must be experimentally assessed [65, 68]
Manufacturing, potency, and product consistency	Loss of viability, genetic identity, or programmed activity between production and administration	Controlled manufacturing and formulation that preserve both microbial viability and therapeutic function	Identity, purity, viable count, genetic integrity, potency, payload output, shelf‐life stability, and batch‐to‐batch consistency	Product quality should reflect functional potency, not viable count alone [69]
Regulatory and clinical translation	A biologically active engineered strain fails to satisfy quality, safety, containment, or clinically meaningful efficacy requirements	Development under an appropriate live biotherapeutic product/biological‐product framework, with product‐specific CMC, safety, shedding, and clinical evaluation	Target engagement, pharmacodynamic response, adverse events, persistence/shedding, environmental risk, and patient‐relevant endpoints	Engineered living therapeutics should not be assumed to follow the same development pathway as conventional probiotic foods or supplements; clinical benefit must be established independently [61, 69]

### 2.9. Beyond Conventional Live Probiotics: Distinct Microbial Therapeutic Platforms

NGPs extend microbiome‐based intervention beyond microorganisms traditionally used as probiotics by selecting human commensals for defined biological functions [70]. *A. muciniphila* is among the most clinically advanced examples. In a randomized proof‐of‐concept study in overweight or obese insulin‐resistant adults, both live and pasteurized *A. muciniphila* were well tolerated, whereas the pasteurized preparation produced favorable changes in selected metabolic outcomes [71]. However, such findings remain intervention‐ and population‐specific and should not be generalized to other NGP candidates. Other microbiome‐derived organisms present different developmental challenges. For example, *F. prausnitzii* is highly sensitive to oxygen; recent work has shown that adaptation and microbial cross‐feeding can improve its manufacturability, with a proof‐of‐concept formulation subsequently evaluated for tolerability in humans [72]. *Blautia wexlerae* has also emerged as a candidate on the basis of human microbiome associations and experimental metabolic effects, although therapeutic efficacy has so far been demonstrated mainly in preclinical models rather than clinical intervention trials [73].

Importantly, a therapeutic effect does not always require administration of a viable microorganism. Pasteurized *A. muciniphila* illustrates that selected biological activity can persist after microbial inactivation [71]. Experimental studies have further identified specific microbial‐derived effectors. The outer‐membrane protein Amuc_1100 has been linked to epithelial‐barrier and regenerative signaling in cell and animal models [74], whereas extracellular vesicles derived from *A. muciniphila* have influenced tight‐junction function and intestinal permeability in experimental systems [75]. These observations broaden the range of microbiome‐based interventions, but the categories should remain distinct. Under the ISAPP framework, postbiotics are preparations of inanimate microorganisms and/or their components that confer a demonstrated health benefit; isolated proteins, vesicles, or other microbial‐derived effectors should therefore not automatically be labeled as probiotics or postbiotics without meeting the relevant definitional criteria [6].

Platform selection should consequently be guided by the function required from the intervention rather than by an assumption that a live microorganism is always preferable. Functions that depend on active sensing, adaptive responses, ecological interactions, or continuous in situ production require viable organisms, including engineered living therapeutics discussed above [61]. In contrast, when the intended effect can be reproduced by an inactivated microbial preparation or a defined microbial‐derived component, a nonlive platform may avoid some of the viability and containment requirements associated with living products. These approaches should therefore be considered related but scientifically distinct therapeutic platforms, with different requirements for characterization, potency, manufacturing, safety, and regulatory evaluation (Figure 4).

**Figure 4:**
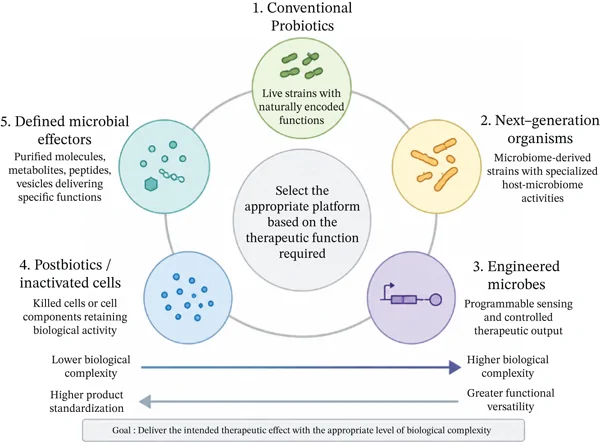
Function‐first selection of microbial therapeutic platforms.

### 2.10. Precision Probiotics: Matching Microbial Function to the Responsive Host

Precision probiotic strategies aim to move beyond population‐average responses by considering whether a particular microbial intervention is suited to the biological context of the recipient [76]. Baseline microbiome composition, microbial functional capacity, host metabolic and immune characteristics, diet, medication exposure, and disease phenotype may all contribute to differences in treatment response [77]. Human studies support the existence of responder and nonresponder patterns, but they also show that prediction is not straightforward. In nonconstipated IBS, a post hoc analysis of a randomized trial with *Lacticaseibacillus paracasei* DG found that responders differed from nonresponders in several baseline microbial features, with *Collinsella aerofaciens* emerging as a candidate predictor of response [78]. Similarly, an analysis of a randomized trial in diarrhea‐predominant IBS identified *Enterococcus faecalis* and *Lactococcus lactis* as potential markers of nonresponse to a multistrain probiotic formulation [79]. These findings provide clinically relevant examples of microbiome‐guided stratification, but neither signature should yet be regarded as a universally validated biomarker.

The limitations of response prediction are equally important. In a randomized probiotic intervention for metabolic syndrome, distinct responder and nonresponder groups were observed, yet baseline microbiome composition and alpha diversity did not reliably predict who would respond [37]. This illustrates why retrospective associations are insufficient for clinical implementation. Candidate biomarkers require prospective validation in independent populations, reproducible analytical methods, and evidence that biomarker‐guided treatment performs better than nonstratified probiotic use [80]. Current precision‐probiotic research therefore remains an emerging clinical strategy rather than an established patient‐selection approach. Multiomics and computational models may strengthen future stratification by combining microbial, metabolic, dietary, and host information, but their value will depend on external validation and prospective testing [5]. A practical precision‐probiotic pathway should therefore begin with identification of the biological dysfunction to be modified, followed by selection of an intervention with an appropriate functional capacity, assessment of host and microbiome features that may influence response, and confirmation of target engagement and patient‐level benefit (Figure 5). In this framework, microbiome signatures are not therapeutic endpoints in themselves; their clinical value depends on whether they can reproducibly identify individuals who are more likely to benefit from a defined intervention.

**Figure 5:**
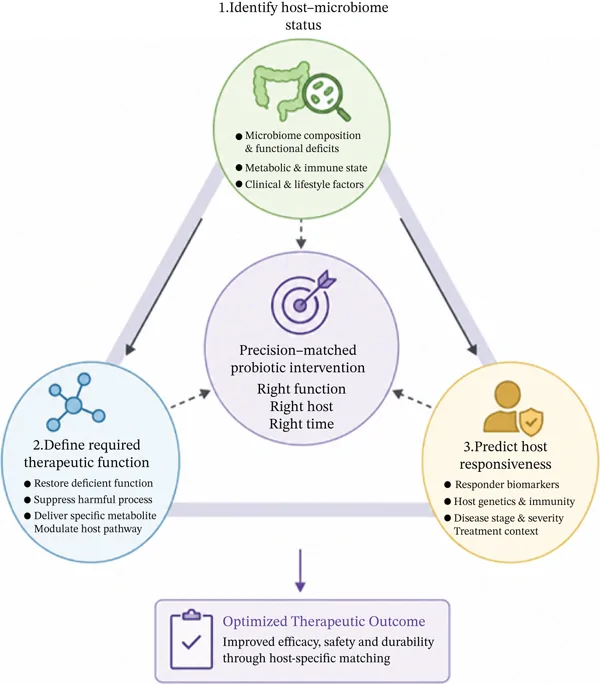
Function‐first framework for precision‐matched probiotic intervention.

### 2.11. Translational and Regulatory Barriers to NGP Therapy

The translation of microbiome‐based therapeutics becomes increasingly complex as development moves beyond conventional probiotics toward next‐generation microorganisms, defined microbial consortia, engineered living therapeutics, and nonlive microbial preparations [81]. These platforms differ in product identity, mechanism of action, manufacturing requirements, potency assessment, and safety profile and therefore should not be considered a single therapeutic or regulatory category [82]. For LBPs, enumeration of viable microorganisms remains an important quality measure, but viable counts alone may not adequately describe potency when therapeutic activity depends on a specific metabolic function, interactions among constituent strains, or another mechanism‐linked biological property. In such cases, viability testing may need to be complemented by appropriately validated functional assays together with assessment of identity, purity, stability, and batch consistency [67, 83]. The principal platform‐specific requirements are summarized in Table 7.

**Table 7:** Platform‐specific quality, potency, and safety considerations for microbiome‐based therapeutics.

Therapeutic platform	Critical product attributes	Potency or functional assessment	Principal safety considerations	Key translational requirement
Conventional probiotic	Confirmed strain identity, viable dose, purity, and stability	Viable count with strain‐ and indication‐specific characterization	Contamination, pathogenic potential, and relevant antimicrobial‐resistance characteristics	Evidence linking the defined strain and administered dose to the intended health benefit [10]
Next‐generation live microorganism	Strain identity, viability, phenotypic stability, and intended biological function	Viability with mechanism‐linked functional assessment where appropriate	Strain‐specific safety, host interaction, persistence, and unintended ecological effects	Reproducible manufacture while preserving the intended functional phenotype [67, 72]
Defined microbial consortium	Identity and quantity of individual members and stability of consortium composition	Member‐specific viable counts with consortium‐level functional testing where relevant	Contamination, loss or overgrowth of individual members, and unintended interstrain interactions	Consistent composition and functional performance across batches [67]
Engineered living therapeutic	Chassis identity, genetic construct, viability, genetic stability, and programmed function	Measurement of the engineered biological output together with microbial viability	Genetic instability, HGT, shedding, environmental persistence, and containment failure	Reproducible programmed activity with appropriate genetic‐stability and biocontainment assessment [61, 65]
Postbiotic/inactivated microbial preparation	Defined microbial source, composition, inactivation process, and retained functional components	Assay linked to the biological activity responsible for the demonstrated benefit	Batch variability, incomplete inactivation where absence of viable organisms is required, and unintended biological activity	Reproducible preparation with retention of the activity associated with the demonstrated health benefit [6]
Microbial‐derived component or defined effector	Molecular or compositional identity, purity, and biological activity	Product‐specific biochemical or functional assay	Toxicity, immunogenicity, or off‐target activity, as applicable	Appropriate product classification and development according to the characteristics of the final therapeutic product [6]

Safety assessment should likewise reflect the characteristics of the final product. Naturally occurring next‐generation microorganisms require strain‐level characterization and evaluation of properties relevant to their intended use, whereas defined microbial consortia additionally require control of individual members and overall community consistency. Engineered living therapeutics introduce further considerations, including genetic stability, horizontal gene transfer, microbial shedding, environmental persistence, and the reliability of biocontainment strategies [61, 65]. In contrast, postbiotics are preparations of inanimate microorganisms and/or their components that confer a demonstrated health benefit and therefore represent a scientifically distinct category from viable probiotics [6]. Purified microbial molecules or isolated effectors should not automatically be classified as probiotics or postbiotics.

Regulatory requirements also vary across jurisdictions, reflecting differences in product classification and existing medicinal‐product frameworks [67]. In the United States, the US Food and Drug Administration (FDA) provides dedicated chemistry, manufacturing, and control guidance for LBPs investigated under an Investigational New Drug application. The guidance applies to early clinical development of LBPs but explicitly excludes microorganisms intended as gene‐therapy vectors, oncolytic bacteria, and oncolytic viruses from its scope [83]. In Europe, the European Pharmacopoeia has established specific quality standards for LBPs, including the general monograph live biotherapeutic products for human use (3053) and general Chapters 2.6.36 and 2.6.38 addressing microbiological examination of these products [84]. The European Medicines Agency (EMA) has also recognized the distinct nonclinical challenges associated with microbiome‐based medicinal products (MBMPs). In 2026, the EMA published a draft concept paper proposing the development of a reflection paper to support a more harmonized approach to their nonclinical evaluation; this initiative remains under development and should not be interpreted as a finalized microbiome‐specific guideline [85].

In the United Kingdom, the MHRA considers MBMPs within the existing medicines regulatory framework. Its 2026 position paper encompasses medicinal products whose principal therapeutic effect is mediated through the microbiome and whose active substances may include live microorganisms, defined microbial consortia, complex microbial ecosystems, or nonviable microorganisms and their derived components. Depending on their characteristics, such products may be regulated as biological medicinal products or, where the relevant legal criteria are met, as advanced therapy medicinal products. The MHRA further emphasizes product characterization, CMC standardization, antimicrobial‐resistance risk, clinically relevant evidence, and early regulatory engagement [86]. A comparison of the principal regulatory approaches is provided in Table 8.

**Table 8:** Regulatory frameworks for microbiome‐based medicinal products.

Jurisdiction/authority	Regulatory approach	Scope/product context	Key development considerations
United States – FDA	Provides dedicated CMC guidance for LBPs investigated under the IND framework	Therapeutic LBPs entering early clinical trials; the specific guidance excludes microorganisms intended as gene‐therapy vectors and oncolytic bacteria	Identity, purity, potency, manufacturing controls, viability, stability, and phase‐appropriate product characterization. [83]
European Union – EMA/European Pharmacopoeia	Microbiome‐based medicinal products are addressed within the medicinal‐product framework; EMA is developing more specific nonclinical guidance, whereas the Ph. Eur. provides dedicated LBP quality standards	LBPs and other microbiome‐based medicinal products according to their product characteristics and intended use	Product‐specific quality and nonclinical assessment; Ph. Eur. monograph 3053 and Chapters 2.6.36 and 2.6.38 provide LBP‐specific quality requirements [84, 85]
United Kingdom – MHRA	MBMPs are regulated within the existing UK medicines framework, with classification determined by product characteristics and intended therapeutic use	May include live microorganisms, defined consortia, complex microbial ecosystems, or nonviable microorganisms and their derived components	Product characterization, CMC, batch consistency, AMR and other product‐specific safety risks, clinical evidence, and early regulatory engagement [86]

Abbreviations: AMR, antimicrobial resistance; CMC, chemistry, manufacturing and controls; EMA, European Medicines Agency; FDA, US Food and Drug Administration; IND, Investigational New Drug; MBMP, microbiome‐based medicinal product; MHRA, Medicines and Healthcare products Regulatory Agency; Ph. Eur., European Pharmacopoeia.

### 2.12. Safety Considerations in Vulnerable Populations and Long‐Term Microbiome Effects

The safety of NGPs deserves particular attention in individuals with weakened immune systems and those receiving critical care. Although probiotics are generally well tolerated, bloodstream infections linked to probiotic microorganisms have occasionally been reported, particularly in patients with compromised intestinal barriers or central venous catheters [87, 88]. These concerns highlight the importance of selecting appropriate strains and assessing patient‐specific risks before administration. Beyond immediate safety, the effects of prolonged probiotic use on the gut microbiome also require consideration. Human studies have shown that microbial colonization varies between individuals and that certain probiotic preparations may interfere with the recovery of native gut microbiota following antibiotic treatment [89]. Whether these changes persist or have meaningful long‐term health consequences remains uncertain. Longer follow‐up studies are therefore needed to evaluate microbial stability, potential adverse effects, and the overall safety of NGP interventions.

### 2.13. Future Perspectives: Toward Precision and Programmable Microbial Medicine

The next stage of microbiome‐based therapeutics is likely to depend less on identifying additional strains and more on determining which microbial function is required, in which biological context, and how that function can be measured after administration [90]. Multiomics approaches may help define host–microbiome states associated with treatment response, whereas computational models can integrate microbial, metabolic, dietary, and host features to support more rational selection of candidate interventions [91]. However, these approaches remain primarily investigational, and their clinical value will depend on reproducibility, external validation, and prospective evidence that model‐guided selection improves patient outcomes [5].

Artificial intelligence may contribute to this process by identifying patterns across high‐dimensional microbiome datasets, prioritizing microbial functions, and developing predictive models of treatment response. Its usefulness will depend on the quality and representativeness of the underlying datasets, standardized analytical pipelines, transparent model validation, and performance across independent populations. AI should therefore be regarded as a tool for improving prediction and experimental prioritization rather than as a substitute for biological or clinical validation [92].

A complementary development is spatial microbiomics, which can move microbiome analysis beyond stool‐level abundance toward the anatomical organization of microorganisms and their interaction with nearby host cells. Spatial host–microbiome sequencing has already demonstrated that microbial geography can be analyzed together with regional host transcriptional responses, although the current evidence is largely experimental and preclinical [93]. Such approaches may eventually help determine not only whether a therapeutic microorganism is present but whether it reaches the biological niche in which its intended function is required.

Human‐relevant experimental models will also be important for narrowing the gap between mechanistic activity and clinical translation. Intestinal organoids can reproduce several epithelial features of the human gut and provide controlled systems for examining host–microbe interactions, barrier responses, and candidate microbial functions [94]. Integration of organoids with immune components, microfluidic systems, and patient‐derived models may improve preclinical evaluation, but these systems should complement rather than replace clinical testing.

Synthetic and systems biology provide a further route toward programmable living therapeutics. Engineered microorganisms can be designed with sense‐and‐respond circuits that detect defined environmental signals and trigger therapeutic outputs, offering the possibility of more spatially and temporally controlled activity [95]. Nevertheless, most such systems remain at the preclinical or early translational stage, and progress will depend on demonstrating genetic stability, reliable in vivo function, appropriate biocontainment, reproducible manufacturing, and clinically meaningful target engagement rather than increasing circuit complexity alone [96].

Together, these technologies suggest a future in which microbial therapeutics are selected and evaluated through an integrated sequence of functional identification, host stratification, spatial target assessment, human‐relevant preclinical validation, and controlled therapeutic delivery. These priorities are summarized in Figure 6. The value of increasingly sophisticated technologies will ultimately depend on whether they improve the predictability, safety, and clinical effectiveness of microbiome‐based interventions.

**Figure 6:**
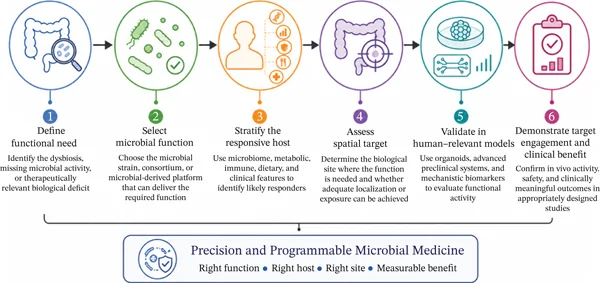
Function‐first translational framework for precision and programmable microbial therapeutics.

### 2.14. Cross‐Cutting Translational Challenges

Despite advances in microbial therapeutic design, successful translation still depends on several interconnected biological and clinical factors. Probiotic effects can be strain‐ and indication‐specific, making results from one organism or disease context difficult to generalize to another [2]. Oral interventions must also retain sufficient activity during gastrointestinal transit, whereas host microbiome composition, diet, metabolic state, and other individual characteristics can influence whether a measurable response occurs [21, 37].

Importantly, microbial detection, survival, or persistence should not be interpreted as evidence of therapeutic efficacy on their own. Translation requires demonstration that the intervention performs the intended biological function in vivo, produces measurable target engagement, and ultimately improves a clinically relevant outcome. This distinction is particularly important because changes in microbial abundance or biomarkers may indicate biological activity without establishing patient benefit. Engineered living therapeutics introduce additional challenges, including maintenance of programmed function, genetic stability, and biosafety during development and clinical use [39, 61, 83]. These barriers are interdependent: An intervention with strong mechanistic activity may still fail clinically because of inadequate delivery, an unsuitable host environment, loss of engineered function, or absence of meaningful target engagement. Table 9 therefore summarizes the principal translational failure points and the evidence needed to address them.

**Table 9:** Major cross‐cutting translational challenges in microbiome‐based therapeutic development.

Domain	Major challenge	Translational consequence	Evidence or development requirement
Strain and functional specificity	Biological and clinical effects vary between strains and indications	Findings from one strain or disease setting may not be transferable to another	Precise strain identification with indication‐specific evidence and mechanism‐relevant characterization [2]
Mechanistic validation	Activity observed in vitro or in animal models may change under human physiological conditions	Mechanistic plausibility may not translate into functional activity in patients	Validation under physiologically and disease‐relevant conditions, followed by confirmation of in vivo target engagement [8, 39]
Delivery and functional exposure	Processing, storage, gastrointestinal conditions, and colonization resistance may reduce the amount of functionally active microorganism reaching the intended site	Delivered dose may not correspond to biologically active exposure	End‐to‐end assessment of viability, delivery, release, and mechanism‐relevant activity [36]
Site‐specific functional activity	Detection or persistence of an intervention at a biological site does not necessarily demonstrate therapeutic function	Microbial presence may be mistaken for successful target engagement	Direct assessment of local functional activity and downstream biological response rather than residence alone [21, 36]
Host heterogeneity	Baseline microbiome, diet, metabolic state, medications, immune characteristics, and disease phenotype may modify response	Responder and nonresponder patterns can reduce population‐level reproducibility	Prospective evaluation and independent validation of candidate responder markers or stratification approaches [38, 37]
Engineered function	Genetic instability, metabolic burden, or variable expression may alter programmed therapeutic activity	Engineered output may decline, fluctuate, or fail under in vivo conditions	Quantitative assessment of genetic stability, functional output, and performance under host‐relevant conditions [61]
Biosafety	Horizontal gene transfer, unintended persistence, environmental dissemination, or containment failure may occur with engineered living therapeutics	Potential risks to the recipient, resident microbiota, or environment	Product‐specific evaluation of HGT, shedding, persistence, genetic stability, and biocontainment [65]
Target engagement versus clinical benefit	Biomarker or microbiome changes may occur without improvement in patient‐relevant outcomes	Biological activity may be overinterpreted as therapeutic efficacy	Prespecified mechanism‐linked biomarkers together with validated clinical endpoints [39]
Clinical evidence and reproducibility	Variation in strain, dose, formulation, population, comparator, and endpoints contributes to heterogeneous findings	Effect estimates may be difficult to reproduce or generalize	Adequately powered trials with clearly identified interventions, appropriate comparators, standardized outcomes, and independent replication [27–31]

## 3. Conclusion

Microbiome‐based therapeutics are evolving beyond conventional probiotic use toward more precisely defined and platform‐specific interventions. Their clinical value will ultimately depend on reproducible biological activity, appropriate patient selection, product‐specific safety and manufacturing control, and evidence of meaningful clinical benefit. Next‐generation microorganisms, engineered living therapeutics, defined consortia, and nonlive microbial products should therefore be evaluated according to their distinct biological and regulatory characteristics. Stronger human evidence and consistent translational validation will be essential for converting promising microbiome‐based interventions into predictable and clinically useful therapies.

## Author Contributions

S.A.: conceptualization, literature review, writing—original draft, visualization. Y.S.P.: validation, writing—review and editing. P.K.: methodology, supervision, writing—review and editing. B.D.: supervision, writing—review and editing. S.S.: conceptualization, formal analysis, writing—review and editing. H.K.: conceptualization, data curation, literature review. B.P.G.: conceptualization, supervision, writing—review and editing, correspondence.

## Funding

No funding was received for this manuscript.

## Disclosure

All authors have read and approved the final manuscript.

## Ethics Statement

The authors have nothing to report.

## Consent

The authors have nothing to report.

## Conflicts of Interest

The authors declare no conflicts of interest.

## Supporting information


**Supporting Information** Additional supporting information can be found online in the Supporting Information section. Table S1: Database‐specific literature search strategies used for the narrative review, including the search terms and search syntax applied in PubMed/MEDLINE, Scopus, Web of Science Core Collection, ScienceDirect, and Google Scholar.

## Data Availability

The authors have nothing to report.
