# Supplementary material for: Next‐Generation Probiotics: Emerging Mechanisms, Therapeutic Innovations, and Future Perspectives
Source: Biomed Res Int. 2026 Nov 14;2026:8353908. doi: 10.1155/bmri/8353908 (PMC13769044; doi:10.1155/bmri/8353908)
Supplement: Supplementary file 1 [file BMRI-2026-8353908-s001.docx]

**Supplementary Table 1. Database-specific search strategy**

| **Database** | **Search strategy** |
| --- | --- |
| **PubMed/MEDLINE** | ("Probiotics"[MeSH Terms] OR probiotic*[Title/Abstract] OR "next-generation probiotic"[Title/Abstract] OR "live biotherapeutic product"[Title/Abstract] OR "engineered probiotic"[Title/Abstract] OR postbiotic [Title/Abstract]) AND ("gut microbiota"[Title/Abstract] OR "intestinal barrier"[Title/Abstract] OR "immune modulation"[Title/Abstract] OR engraftment[Title/Abstract] OR "clinical efficacy"[Title/Abstract] OR responder*[Title/Abstract] OR "microbial delivery"[Title/Abstract] OR "synthetic biology"[Title/Abstract] OR "multi-omics"[Title/Abstract] OR safety[Title/Abstract] OR regulation[Title/Abstract]) |
| **Scopus** | TITLE-ABS-KEY(probiotic OR "next-generation probiotic" OR "live biotherapeutic product" OR "engineered probiotic" OR postbiotic) AND TITLE-ABS-KEY("gut microbiota" OR "intestinal barrier" OR "immune modulation" OR engraftment OR "clinical efficacy" OR responder OR "microbial delivery" OR "synthetic biology" OR "multi-omics" OR safety OR regulation) |
| **Web of Science Core Collection** | TS=(probiotic* OR "next-generation probiotic" OR "live biotherapeutic product" OR "engineered probiotic" OR postbiotic*) AND TS=("gut microbiota" OR "intestinal barrier" OR "immune modulation" OR engraftment OR "clinical efficacy" OR responder* OR "microbial delivery" OR "synthetic biology" OR "multi-omics" OR safety OR regulation) |
| **ScienceDirect** | Focused combinations of terms related to probiotics, next-generation probiotics, engineered probiotics, postbiotics, mechanisms, clinical efficacy, delivery, safety, and regulation. |
| **Google Scholar** | Targeted phrase searches using combinations such as “next-generation probiotics” AND efficacy, “engineered probiotics” AND clinical evidence, “probiotic engraftment” AND host microbiome, and “live biotherapeutic products” AND regulation. |
